# A case of prolonged exertional rhabdomyolysis in a MHS individual

**DOI:** 10.1186/1471-2253-14-S1-A6

**Published:** 2014-08-18

**Authors:** B Kraft, R Hahn, K Markstaller, HG Kress

**Affiliations:** 1 Department of Special Anaesthesia and Pain Therapy, Medical University of Vienna, Vienna, 1090, Austria; 2 Division of General Anaesthesia and Intensive Care Medicine, Medical University of Vienna, Vienna, 1090, Austria

## Background

We report a case of prolonged exercise-induced rhabdomyolysis (ER) in an otherwise healthy male malignant hyperthermia susceptible (MHS) individual.

## Case report

A 42-year old healthy Caucasian male contacted our malignant hyperthermia (MH) hotline due to cramps and muscle pain one week after moderate exercise. He was already diagnosed MHS in 1988 by in vitro contracture test (IVCT), after a suspected MH episode during general anaesthesia.

At present, he reported severe muscle pain and cramps in both upper legs. The discomfort has started after moderate endurance training with mild muscle soreness for 2 days. On the 3^rd^ day, pain increased, accompanied by severe muscle cramps in both upper legs. Symptoms were worsened by cold temperature. Due to pain, muscle weakness and swelling in both upper legs, he had sought medical attention on day 5 in a surgical outpatient clinic, where an ultrasound examination showed a “homogenous increase in muscle density of both quadriceps muscles”. No laboratory tests except d-dimer were performed and after exclusion of an acute thromboembolic event, the patient was dismissed. Due to ongoing pain, he contacted our MH-Hotline 7 days after the exercise. He still felt sick and reported darker urine during the past 3 days. Body temperature was not yet measured. His general practitioner had already taken a blood examination one hour before the telephone call. One day later, laboratory analysis was available and showed a CPK of 39,628 U/l and myoglobin of 2,863 ng/ml, consistent with rhabdomyolysis. Also liver enzymes were elevated, but kidney function and potassium were normal. The patient reported a body temperature of 37.5°C in the morning. Due to the high CPK levels 7 days after exercise (normally, peak levels are expected within 48 to 96 h), we recommended an immediate admission to our unit and decided to start with dantrolene 2.5 mg/kg IV. After the initial dose of dantrolene he was admitted to an intermediate care unit for the first 24h. Dantrolene was not continued since he was clinically stable. No clinical signs of a compartment syndrome could be detected. Pain continuously decreased and laboratory parameters slowly returned to normal within the next 2 weeks except of a CPK of 330 U/l. Body temperature and kidney function remained normal during the whole period. Any other reason for rhabdomyolysis than MHS diagnosis, i.e. viral or bacterial infection, hypothyreosis, medication, drugs or dietary supplements could be ruled out. A MRI examination showed an inhomogenous enhancement of contrast medium and edema of both quadriceps muscles. The patient was dismissed from hospital 3 days later, additional genetic testing has been planned. Unfortunately, previous CPK levels of the patient were unknown and in 1988, when IVCT was performed, histology was not regularly done.

## Conclusions

With respect to this case, many questions concerning ER remain open. First, there are no guidelines for the acute management and the specific treatment of ER. There is some evidence from the literature supporting initial fluid therapy, but insufficient data exist, whether or to which patients dantrolene should be administered and how much and how long it should be given. There are no parameters defined that indicate how long the patient should be followed.

Secondly, there are only sparse data that provide some guidance on what we should recommend to the patient to avoid further relapses of ER. Finally, no sufficient data exist about the therapeutic value of preventive measures such as oral dantrolene or carnitine phosphate.

